# Arsenic trioxide impacts hepatitis B virus core nuclear localization and efficiently interferes with HBV infection

**DOI:** 10.1128/spectrum.03788-23

**Published:** 2024-04-03

**Authors:** Samuel Hofmann, Julius Luther, Verena Plank, Andreas Oswald, Julia Mai, Ilka Simons, Julija Miller, Valeria Falcone, Lea Hansen-Palmus, Hartmut Hengel, Michael Nassal, Ulrike Protzer, Sabrina Schreiner

**Affiliations:** 1 Institute of Virology, School of Medicine, Technical University of Munich, Munich, Germany; 2 Institute of Virology, Hannover Medical School, Hannover, Germany; 3 Cluster of Excellence RESIST (Resolving Infection Susceptibility, EXC 2155), Hannover Medical School, Hannover, Germany; 4 Division of Pediatric Neurology and Metabolic Medicine, Center for Pediatric and Adolescent Medicine, University Hospital Heidelberg, Heidelberg, Germany; 5 Department of Internal Medicine II/Molecular Biology, University Hospital Freiburg, Freiburg, Germany; 6 Institute of Virology, Medical Center – University of Freiburg, Freiburg, Germany; 7 Institute of Virology, Helmholtz Zentrum München, Munich, Germany; 8 German Center for Infection Research (DZIF), Munich, Germany; University of Arizona, Tucson, Arizona, USA

**Keywords:** hepatitis B virus, HBV, antivirals, arsenic, PML-NB, SUMO, cccDNA, dsDNA viruses

## Abstract

The key to a curative treatment of hepatitis B virus (HBV) infection is the eradication of the intranuclear episomal covalently closed circular DNA (cccDNA), the stable persistence reservoir of HBV. Currently, established therapies can only limit HBV replication but fail to tackle the cccDNA. Thus, novel therapeutic approaches toward curative treatment are urgently needed. Recent publications indicated a strong association between the HBV core protein SUMOylation and the association with promyelocytic leukemia nuclear bodies (PML-NBs) on relaxed circular DNA to cccDNA conversion. We propose that interference with the cellular SUMOylation system and PML-NB integrity using arsenic trioxide provides a useful tool in the treatment of HBV infection. Our study showed a significant reduction in HBV-infected cells, core protein levels, HBV mRNA, and total DNA. Additionally, a reduction, albeit to a limited extent, of HBV cccDNA could be observed. Furthermore, this interference was also applied for the treatment of an established HBV infection, characterized by a stably present nuclear pool of cccDNA. Arsenic trioxide (ATO) treatment not only changed the amount of expressed HBV core protein but also induced a distinct relocalization to an extranuclear phenotype during infection. Moreover, ATO treatment resulted in the redistribution of transfected HBV core protein away from PML-NBs, a phenotype similar to that previously observed with SUMOylation-deficient HBV core. Taken together, these findings revealed the inhibition of HBV replication by ATO treatment during several steps of the viral replication cycle, including viral entry into the nucleus as well as cccDNA formation and maintenance. We propose ATO as a novel prospective treatment option for further pre-clinical and clinical studies against HBV infection.

**IMPORTANCE:**

The main challenge for the achievement of a functional cure for hepatitis B virus (HBV) is the covalently closed circular DNA (cccDNA), the highly stable persistence reservoir of HBV, which is maintained by further rounds of infection with newly generated progeny viruses or by intracellular recycling of mature nucleocapsids. Eradication of the cccDNA is considered to be the holy grail for HBV curative treatment; however, current therapeutic approaches fail to directly tackle this HBV persistence reservoir. The molecular effect of arsenic trioxide (ATO) on HBV infection, protein expression, and cccDNA formation and maintenance, however, has not been characterized and understood until now. In this study, we reveal ATO treatment as a novel and innovative therapeutic approach against HBV infections, repressing viral gene expression and replication as well as the stable cccDNA pool at low micromolar concentrations by affecting the cellular function of promyelocytic leukemia nuclear bodies.

## INTRODUCTION

Hepatitis B virus (HBV) is a small, enveloped partially double-stranded (ds) DNA virus containing a relaxed circular DNA (rcDNA) genome (1, 2). Overall, this viral genome encodes four different open reading frames responsible for the transcription of the core, surface, polymerase, and the X protein (HBx) (1, 3–6).

HBV infection is initiated by the interaction of the viral particle with heparan sulfate proteoglycans and the sodium taurocholate cotransporting polypeptide (NTCP) receptor (7, 8). Subsequently, the viral inner nucleocapsid, consisting of 120 core protein dimers and harboring the rcDNA, is released into the cytoplasm and transported to the nucleus (1, 2, 9–11). Following the transfer of rcDNA into the nucleus, where it mimics damaged cellular DNA, several host cellular repair mechanisms convert the rcDNA into covalently closed circular DNA (cccDNA) (1, 12). This cccDNA minichromosome serves as the transcriptional template for pregenomic RNA (pgRNA) and subgenomic RNAs and ensures the persistence of HBV infection (1). The pgRNA is reverse transcribed into rcDNA within *de novo*-formed capsids consisting of core proteins. Those mature capsids are enveloped at the endoplasmic reticulum and released via multivesicular bodies as novel-formed virions (13, 14). A second alternative pathway for mature capsids is recirculation, implying their redirection toward the nucleus to establish the stable cccDNA pool and ensure HBV persistence (15, 16).

Despite a preventive vaccine, still over 296 million individuals are chronically infected with HBV worldwide and around 820,000 people die annually due to chronic HBV-related diseases like liver cirrhosis or hepatocellular carcinoma (HCC) (17), with HCC being one of the leading causes of cancer-related deaths worldwide (18). Current treatment strategies against HBV solely block the progression of infection and combat symptoms but do not provide a curative ultimate clearance of the virus. These clinical therapy approaches are mainly based on the administration of life-long treatment with PEGylated interferon alpha2a and/or nucleos(t)ide analogs (NAs) like lamivudine, adefovir, entecavir, telbivudine, or tenofovir (19, 20). Novel therapeutic approaches include the development of direct-acting antivirals. Capsid assembly modulators, small molecules that directly bind HBV core proteins to either prevent or accelerate their assembly, were shown to block cccDNA amplification by yielding empty, aberrant, or non-infective HBV nucleocapsids but cannot interfere with established cccDNA pools [reviewed in reference (21)].

A further direct-acting approach is Bulevirtide (formerly Myrcludex B) blocking virus entry by interference with HBV and HDV binding to the NTCP receptor, which shows promising results in clinical trials but is also not able to directly affect HBV cccDNA (22–26). Taken together, these studies emphasize the urgent need for novel, innovative, and potent therapeutic treatment options that clear or at least partially affect the persistence reservoir of HBV, the stable cccDNA.

Recent studies from our lab established a link between the HBV core protein, its post-translational modification with the small ubiquitin-related modifier SUMO, subsequent association with promyelocytic leukemia nuclear bodies (PML-NBs), and generation of cccDNA from its rcDNA precursor. We could hereby demonstrate that SUMOylation of the HBV core protein is a prerequisite for the disassembly of HBV nucleocapsids, nuclear entry, and the interaction with PML-NBs and that these mechanisms, as well as PML-NBs *per se*, play a pivotal role in cccDNA generation.

PML-NBs are matrix-associated, nuclear dot-like multiprotein complexes (27–29). The major constitutive components of PML-NBs are the PML proteins, Sp100, Daxx, and SUMO, while PML-associated proteins are dynamically recruited by interaction with and post-translational modifications by SUMO (29, 30). PML number, function, and composition vary depending on the cell type, cell cycle stages, and cellular stress response (31, 32). Thereby, PML-NBs obtain a wide range of different functions, participating in various cellular processes like senescence, apoptosis, transcription, replication and epigenetic silencing, protein degradation, antiviral defense, and DNA damage response, dependent on the protein composition of the PML-NBs (33–43). Various viruses, like human adenovirus and herpesviruses, target PML-NBs to specifically interfere with these antiviral defense mechanisms (34, 44–46). In contrast, viral genomes and replicational processes are often associated with PML-NBs, indicating also the beneficial impact of PML-associated components for viral augmentation (47, 48). During HBV infection, earlier studies suggested an interaction of the HBV core protein with PML-NBs in cell lines producing HBV in which cellular stress was induced by DNA-damaging agents (49). Work from our group further deepened the understanding of the HBV core protein/PML interplay by showing that the HBV core/PML interaction depends on the SUMOylation of HBV core protein and that SUMOylation of HBV core mediates nuclear entry and PML association of incoming capsids, all in all enhancing cccDNA formation and efficient HBV replication (48).

Our group recently published that infection of human adenoviruses (HAdV), which are known to extensively interact with PML-NBs [reviewed in reference (50)], can be potently inhibited by treatment with arsenic trioxide (ATO/As_2_O_3_), mainly by interference with HAdV-induced PML-NB relocalization, disruption, and deregulation of the cellular SUMO pool (51).

ATO is an ancient therapeutic compound that was already used in traditional Greek and Chinese medicine over 2,000 years ago. In 1992, ATO was first described to induce efficient and complete clinical remission in two-thirds of patients suffering from acute promyelocytic leukemia (APL) (52–55). Thereupon, the U.S. Food and Drug Administration approved ATO as a treatment for APL in 2000 (56). In the genetic disorder APL, PML is fused to the retinoic acid receptor alpha, resulting in the aberrant track-like structure of promyelocytic leukemia nuclear bodies, leading to the complete loss of PML-NB functionality (57–59). ATO binds directly to cysteine residues within the PML protein, evoking oxidation of PML and leading to the reformation of the dot-like structure of PML-NBs in APL patient cells (60, 61). Subsequently, PML and PML-NB-associated proteins undergo hyperSUMOylation, followed by ubiquitinylation by the SUMO-dependent E3-ubiquitin ligase RNF4, provoking the proteasomal degradation of these substrates (60–63).

Until now, there have been conflicting results on the impact of ATO and arsenicals on HBV and HCC. A study by Chung and Wu (64) showed that treatment of transgenic HBsAg-expressing mice with ATO induced a decrease in the incidence and size of HCC but could not affect overall survival rates. On the other hand, a link was proposed between the intake of arsenicals, especially mono- and dimethylated organic derivatives of ATO, in contaminated drinking water and the incidence of chronic HBV infection (65), whereas another study reported a beneficial effect of high-arsenic drinking water intake on the prevalence of hepatitis or liver cirrhosis in HBV-positive individuals and even a higher amount of HBV inactivation in those populations (66).

The molecular effect of ATO on HBV infection, protein expression, and cccDNA formation and maintenance, however, has not been characterized and understood until now.

In this study, we reveal ATO treatment as a novel and innovative antiviral approach against HBV infections, repressing viral gene expression and replication as well as the stable cccDNA pool at low micromolar concentrations by affecting the cellular function of PML-NBs.

## RESULTS

### ATO treatment diminishes HBV core protein expression

**Fig 1:**
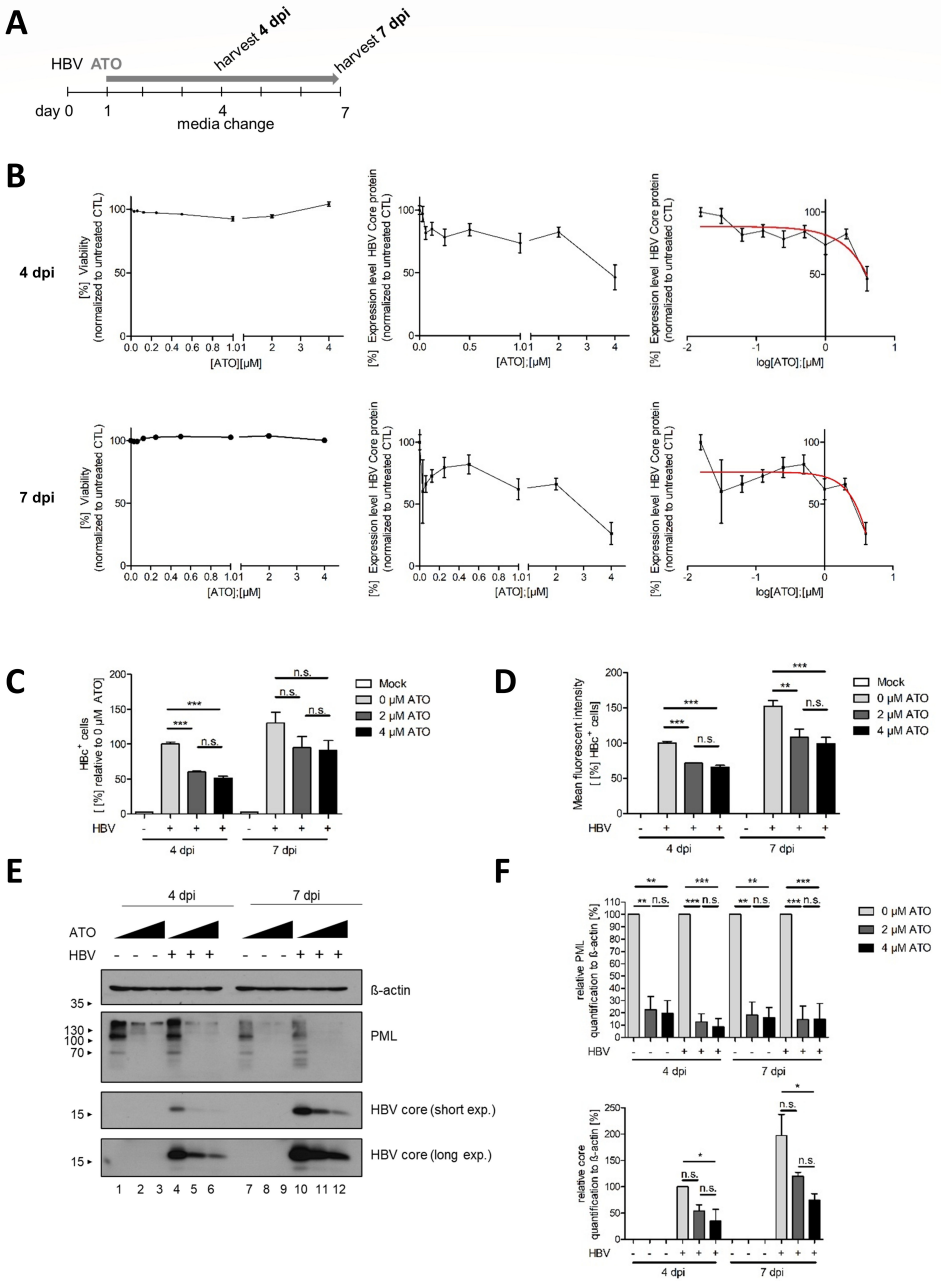
Reduction of HBV infectivity by ATO treatment. HepG2-NTCP-K7 cells were infected with HBV (MOI 200), treated with the indicated concentrations of ATO at 20 hpi, and analyzed at 4 and 7 dpi. (A) Schematic representation of the experimental setup. (B) Cell viability of HBV-infected and ATO-treated cells was assessed using the Promega CellTiter-Blue Cell Viability Assay system prior to fixation with 4% paraformaldehyde and staining with mAb sc-23945 (core). HBV core fluorescence intensity was measured using a Tecan Infinite 200M plate reader at an excitation and emission wavelength of 488 nm. Fluorescence intensity values were normalized to untreated, infected cells. IC50 values were determined by a logarithmic data representation and a fitted logarithmic function represented by a red line in the right panels. Data correspond to six biological replicates. (C) Cells were fixed, stained (rbAb 216A-14-ASR, core), and HBc flow cytometry analysis was performed. Percentage of HBc-positive cells was quantified by FlowJo relative to untreated, infected cells. (D) Mean fluorescence intensity of HBc-positive cells was calculated relative to untreated, HBV-infected cells. (E) Cells were harvested, protein lysates were prepared, separated by SDS-PAGE, and subjected to immunoblotting using mAb 8C9-11 (core), pAb NB100-59787 (PML), and mAb AC-15 (β-actin). Molecular weights are depicted in kDa on the left, and proteins are depicted on the right-hand side, respectively. (F) Protein expression was quantified by densitometric analysis of the detected bands using ImageJ (version 1.45s). Relative protein expression was normalized to β-actin levels. Bar charts represent average values and standard deviations based on three independent experiments. Statistical significance was determined using one-way ANOVA. *P ≤ 0.05, **P ≤ 0.01, ***P ≤ 0.001, and ****P ≤ 0.0001.

Previous pharmacokinetic studies on ATO revealed the therapeutic range upon oral as well as intravenous drug administration from 1 µM up to 7.3 µM as effective concentrations (52, 67). In the first step, the cell viability of HBV-infected HepG2-NTCP-K7 cells during ATO treatment was determined by analyzing a dilution of different compound concentrations at 4- and 7-days post-infection (dpi) (Fig. 1A). In the course of this experiment, no cytotoxic effects utilizing ATO amounts up to 4 µM treatment could be detected (Fig. 1B). Next, the application of different ATO concentrations (0–4 µM) on HBV core protein expression levels was investigated. Administration of 4 µM ATO reduced core levels at 4 dpi to less than 50% compared to untreated controls, indicating a half maximal inhibitory concentration IC50 of 4.64 µM (Fig. 1B). At 7 dpi, 4 µM of ATO application reduced the core protein expression to 30% corresponding to an IC50 of 2.88 µM (Fig. 1B). Based on these data, we concluded to apply concentrations of 2 and 4 µM ATO for all further experiments.

Primarily, HBV core (HBc) expression of infected and ATO-treated HepG2-NTCP-K7 cells at 4 and 7 dpi was analyzed by flow cytometry (Fig. 1C and D). These results showed a significant reduction in HBc^+^ cells following treatment with 2 or 4 µM ATO compared to untreated, infected control cells at 4 dpi (Fig. 1C). The number of core positive cells was significantly reduced after ATO treatment early as well as late after HBV infection. Moreover, the mean fluorescence intensity was calculated, displaying a significant decrease in HBc expression in infected cells after ATO application at both time points (Fig. 1D).

To further validate our previous findings on HBc protein expression, western blot analysis of endogenous PML and the viral core protein was performed. Consistent with earlier reports, the PML protein levels were reduced after ATO application at 4 dpi as well as 7 dpi and served as a treatment control (Fig. 1E and F) (60–63). ATO treatment induced a strong diminution of HBV core protein expression. Core protein expression levels were quantified relatively to β-actin and normalized to untreated controls, showing severe reductions in core protein synthesis (Fig. 1E and F). In summary, HBV core protein expression was decreased by ATO administration at early as well as late time points after HBV infections.

### ATO administration declines HBV replication and transcription

**Fig 2:**
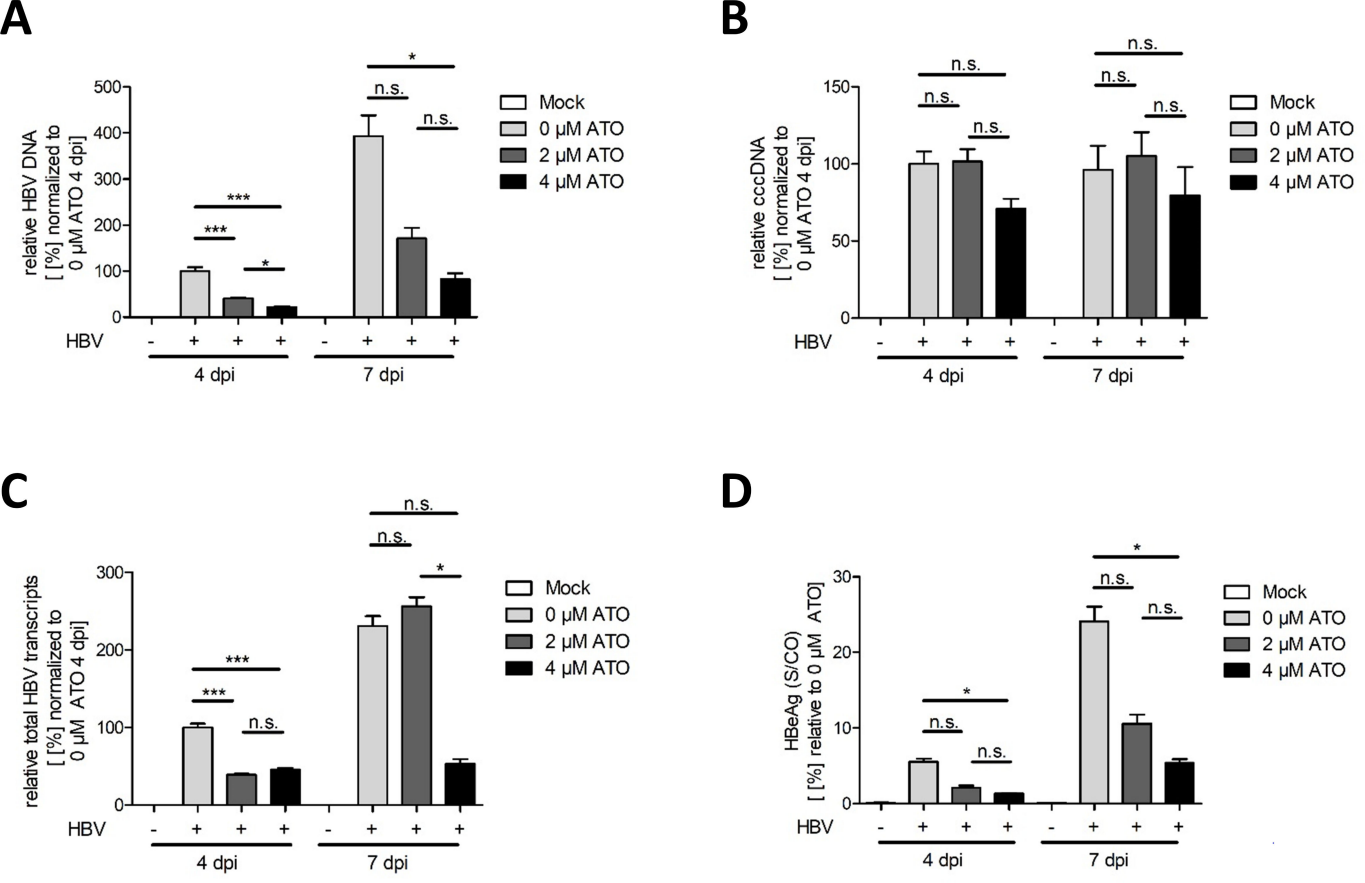
ATO treatment induces diminution of HBV replication and expression. HepG2-NTCP-K7 cells were infected with HBV (MOI 200) and treated with the depicted concentration of ATO at 20 hpi. Cells were subjected to analysis at 4 and 7 dpi. (A) Total DNA was isolated and subjected to RT-PCR using primers specific for the total intracellular HBV DNA relative to PRP as an internal control. Values were normalized to untreated, infected cells at 4 dpi. (B) Quantification of intracellular cccDNA was performed by RT-PCR analysis utilizing specific HBV cccDNA primers relative to PRP as an internal control and normalized to untreated, infected cells at 4 dpi. (C) Total HBV transcript quantification was performed after mRNA extraction by RT-PCR using specific primers. Data were quantified relative to the respective PRP levels and normalized to untreated, infected cells at 4 dpi. (D) HBeAg in cell culture supernatant was measured and normalized to untreated, infected cells. Bar charts represent average values and standard deviations based on six biological replicates. Statistically significant differences were determined using one-way ANOVA. *P ≤ 0.05, **P ≤ 0.01, ***P ≤ 0.001, and ****P ≤ 0.0001.

In the next step, the effect of ATO upon efficient total HBV DNA replication was examined using quantitative real-time PCR (RT-PCR). Cells were infected with HBV, treated with the indicated concentration of ATO, and incubated for 4 and 7 dpi. ATO treatment induced a highly significant reduction of total HBV DNA (Fig. 2A). In line with this, the stable transcriptional HBV template, the cccDNA, was investigated. Our results demonstrated a slight, however, not significant diminution of cccDNA levels after 4 µM ATO treatment, pronounced early after infection (Fig. 2B). To further assay the reduction of total HBV transcripts by ATO, mRNA was extracted, and RT-PCR analysis was performed. Quantifications displayed a significant reduction of total HBV transcripts at 4 dpi, while at 7 dpi, only 4 µM induced a strong reduction in HBV transcription (Fig. 2C). Furthermore, levels of secreted HBeAg from HBV-infected cells treated with ATO were assessed. We observed significant reductions in HBeAg levels during ATO application relative to untreated, infected cells at 4 dpi at early as well as late time points post-infection. Increasing the compound concentration to 4 µM led to an even stronger and also significant reduction of HBeAg values at both time points (Fig. 2D). Finally, ATO treatment diminishes the HBV replication, transcription, and HbeAg secretion.

### ATO treatment decreases the number of PML nuclear bodies and relocalizes HBV core proteins

**Fig 3:**
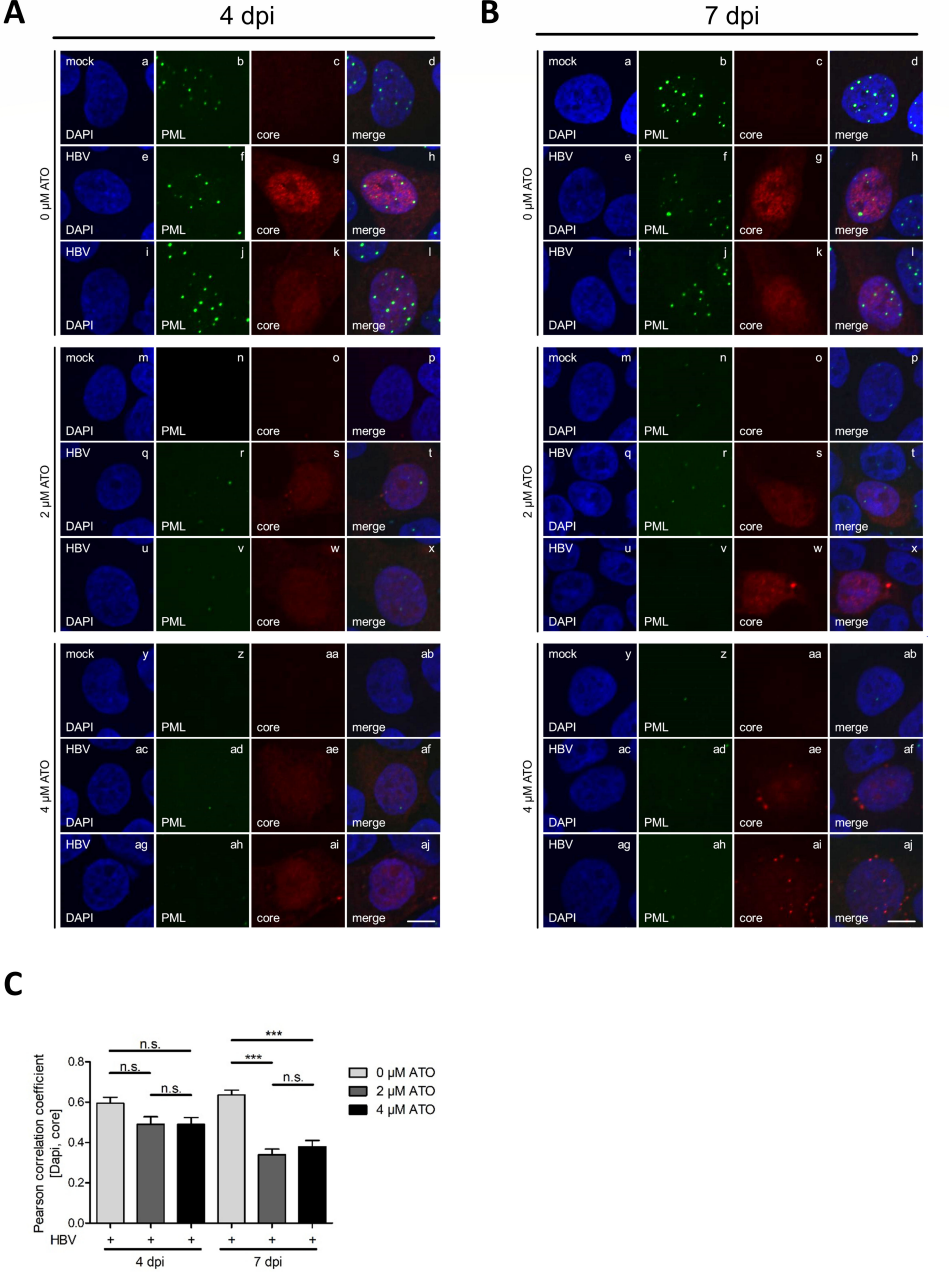
Intracellular HBV core protein localization efficiently vanished during ATO treatment. Differentiated HepG2-NTCP-K7 cells were infected with HBV (MOI 200) and treated with the depicted amount of ATO at 20 hpi. Cells were fixed at 4 and 7 dpi with 4% paraformaldehyde and stained with mAb sc-23945 (core) and pAb NB100-59787 (PML). Primary antibodies were detected by conjugated secondary antibodies Alexa488 (PML, green) and Alexa647 (core, red). (**A**) Representative pictures are shown for cells fixed at 4 dpi. (**B**) Representative pictures are shown for cells fixed at 7 dpi. Scale bar represents 7 µm. (**C**) Nuclear localization of the HBV core was determined using Pearson correlation analysis of the HBV core and DAPI as a nuclear marker. Statistical significance was determined using one-way ANOVA. *P ≤ 0.05, **P ≤ 0.01, ***P ≤ 0.001, and ****P ≤ 0.0001. Data correspond to at least 17 cells.

To substantiate our previous data, the impact of ATO on intracellular PML-NBs as well as HBV core proteins in mock and HBV-infected cells was determined via immunofluorescence studies. According to acquainted literature, our stainings displayed a strong decrease of PML-NB numbers in mock and infected cells at 4 and 7 dpi after ATO treatment (Fig. 3A and B) (60–63). Diffuse localization of HBV core mainly in the nucleus as well as the cytoplasm in untreated cells corresponded to known and published data (Fig. 3A and B, panels e–l) (68–70). Moreover, our immunofluorescence stainings indicate a reduced signal intensity for the HBV core after 2 µM (Fig. 3A and B, panels q–x) and 4 µM (Fig. 3A and B, panels ac–aj) ATO administration. Particularly, the nuclear core staining declined after ATO treatment. Infected cells only showed a slight cytoplasmic relocalization for the HBV core between varying ATO concentrations at 4 dpi. Cells fixed after 7 dpi also exhibited reduced core staining intensity as observed for 4 dpi; however, additionally, less nuclear core localization was detectable in cells treated with 2 µM ATO (Fig. 3B, panels q–x). Application of 4 µM ATO not only showed decreased nuclear core localization but also accumulation in cytoplasmic dots (Fig. 3B, panels ac–aj). This ATO-induced loss of nuclear core protein localization could further be confirmed by Pearson correlation analysis of the colocalization of HBV core and DAPI as a nuclear marker, showing a significant redistribution into the cytoplasm at 7 dpi in ATO-treated cells (Fig. 3C). Synoptically, application of ATO induced decreased PML-NB as well as HBV core levels during immunofluorescence analysis and a relocalization of the HBV core, including nuclear exclusion and accumulation in the cytoplasm.

### ATO significantly decreases HBV protein expression after the establishment of an HBV infection

**Fig 4:**
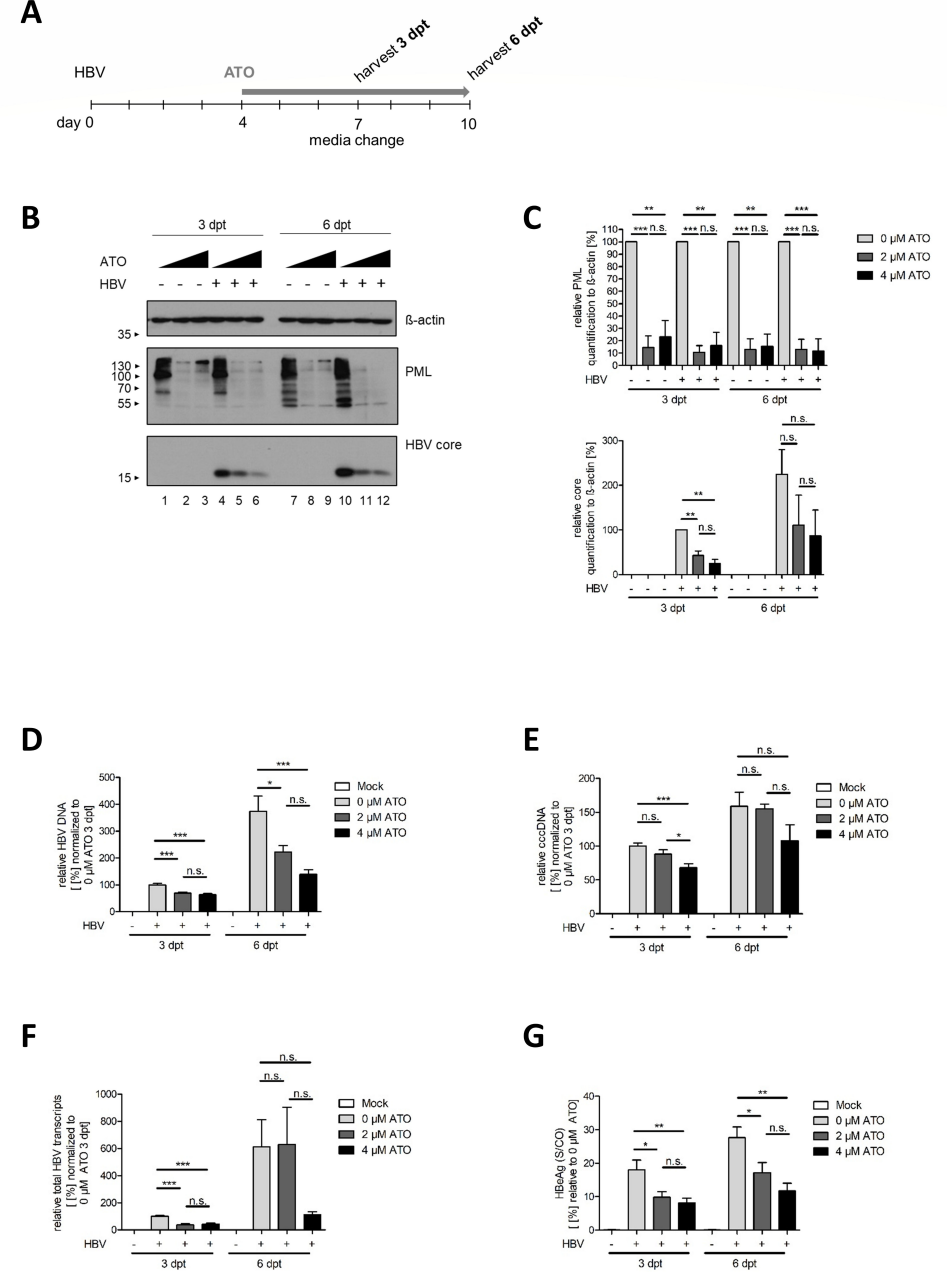
ATO treatment efficiently diminishes established HBV infection. Differentiated HepG2-NTCP-K7 cells were infected with HBV (MOI 200) and treated at 4 dpi with the indicated amount of ATO. Samples were harvested and analyzed at 3 and 6 dpt. (A) Schematic representation of the experimental setup. (B) Cells were harvested, whole-cell lysates were prepared, separated by SDS-PAGE, and subjected to immunoblotting using mAb 8C9-11 (core), pAb NB100-59787 (PML), and mAb AC-15 (β-actin). Molecular weights in kDa are depicted on the left and respective proteins on the right of each blot. (C) Protein expression of the detected bands was quantified by densitometric analysis utilizing ImageJ (Version 1.45 s). Relative protein expression was normalized to the respective β-actin steady-state level. Bar charts represent average values and standard deviations based on three independent experiments. (D) Cells were subjected to RT-PCR analysis at 3 and 6 dpt using primers for the total intracellular HBV DNA relative to PRP as an internal control, normalized to untreated, infected cells at 3 dpt. (E) Quantification of intracellular cccDNA by RT-PCR analysis was performed respectively to panel A, utilizing specific HBV cccDNA primers. (F) HBV total transcript quantification was performed after mRNA extraction by RT-PCR using primers specific for total HBV transcripts. Data were normalized to the respective PRP levels relative to 4 dpi with 0 µM ATO treatment. (G) Secreted HBeAg quantity in cell culture supernatant was determined by performing an ELISA assay. Results depicted in bar charts represent average values and standard deviations based on six biological replicates. Statistically significant differences were determined using one-way ANOVA. *P ≤ 0.05, **P ≤ 0.01, ***P ≤ 0.001, and ****P ≤ 0.0001.

To assay the impact of ATO on HBV during an established HBV infection, we simultaneously treated HBV-infected cells at 4 dpi, as the cccDNA pool at that time point is already stably established (71), and harvested 3 or 6 days post-treatment (dpt) (Fig. 4A). By performing western blot analysis of endogenous PML and the viral core protein, we were able to substantiate our previous findings. PML was used as a control for efficient ATO treatment, which could be validated by the reduced protein expression levels depicted in Fig. 4B and quantified in Fig. 4C. Moreover, ATO treatment strongly impaired HBV core protein synthesis after short as well as long treatment periods of established HBV infections (Fig. 4B). Additionally, core levels were quantified relatively to β-actin and normalized to untreated controls, showing substantial reductions after ATO administration (Fig. 4C).

### ATO reduces replication, transcription, and HBeAg synthesis in an established HBV infection

To further validate ATO-mediated inhibition of established HBV infection, total HBV DNA was extracted and quantified via RT-PCR analysis. Application of ATO significantly decreased the total HBV DNA levels at 3 and 6 dpt in comparison to untreated, infected controls (Fig. 4D). Furthermore, HBV cccDNA levels were quantified, and it revealed a certain reduction even in established HBV infections when treated with 4 µM ATO (Fig. 4E). Taken together, these results display a strong diminution of total HBV DNA as well as a partial reduction of cccDNA levels post-ATO treatment in an established HBV infection. Moreover, the effect of ATO on the number of total HBV transcripts was evaluated (Fig. 4F). These findings correspond well with our results outlined above, revealing a reduction in total HBV transcripts at 3 dpt as well as 6 dpt, especially for cells treated with 4 µM ATO. To substantiate our previous findings, levels of secreted HBeAg were determined. Figure 4G shows the diminution of HBeAg levels during ATO treatment relative to untreated, infected cells especially early after infection. HBeAg was reduced even stronger with increasing compound concentration, supporting our previous findings. In summary, these results display ATO to even reduce HBV total DNA, total transcripts, HBeAg secretion, and partially cccDNA levels in stable HBV-infected cells.

### ATO relocalizes HBV core during established infection

**Fig 5:**
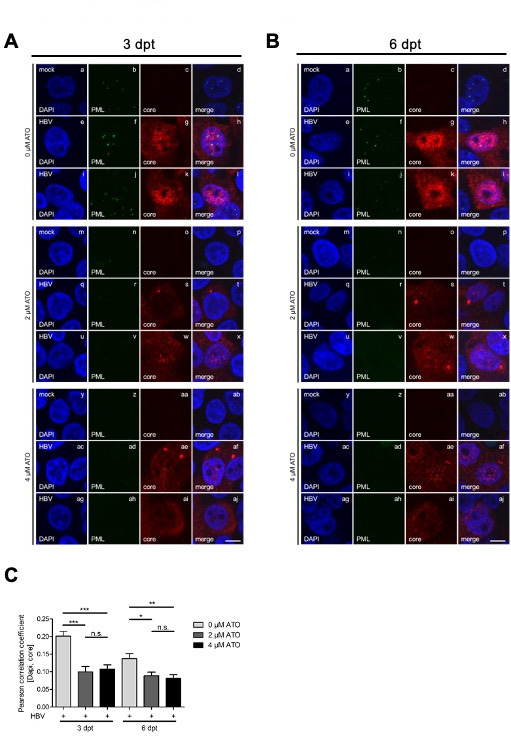
HBV core is substantially relocalized by ATO administration during established HBV infection. Differentiated HepG2-NTCP-K7 cells were infected with HBV (MOI 200) and treated with the depicted amount of ATO at 4 dpi. Cells were fixed at 3 and 6 dpt with 4% paraformaldehyde and stained with mAb sc-23945 (core) and pAb NB100-59787 (PML). Primary antibodies were detected by conjugated secondary antibodies Alexa488 (PML, green) and Alexa647 (core, red). (**A**) Representative immunofluorescence images are shown for cells fixed at 3 dpt. (**B**) Representative pictures are shown for cells fixed at 6 dpt. Scale bar represents 7 µm. (**C**) Nuclear localization of HBV core was determined using Pearson correlation analysis of HBV core and DAPI as a nuclear marker. Statistical significance was determined using one-way ANOVA. *P ≤ 0.05, **P ≤ 0.01, ***P ≤ 0.001, and ****P ≤ 0.0001. Data correspond to at least 100 cells.

We further determined the impact of ATO treatment on the localization of HBV core during established HBV infection. In accordance with the immunofluorescence data for treatment directly after infection (Fig. 3A and B), a decrease in PML-NB number upon treatment with 2 and 4 µM ATO could be observed at 3 and 6 dpt (Fig. 5A and B, panels b, f, j, n, r, v, z, ad, and ah). Additionally, the immunofluorescence stainings confirmed the redistribution of the HBV core protein from a diffuse nuclear and cytoplasmic localization for untreated cells (Fig. 5A and B, panels e–l) to a loss of nuclear localization and an induction of cytoplasmic accumulations during treatment with 2 and 4 µM ATO at 3 and 6 dpt (Fig. 5A and B, panels q–x and ac–aj). , Pearson correlation analysis of the colocalization of core and DAPI as nuclear marker confirmed these findings, showing a significant loss of nuclear core (Fig. 5C). The lower values of HBV core localization to the nuclear DAPI staining compared to Fig. 3 can be explained by the later time point, as HBV core shows a native localization toward the cytoplasm at later time points after infection (72).

### Cessation of ATO treatment induces a relapse of HBV infection

**Fig 6:**
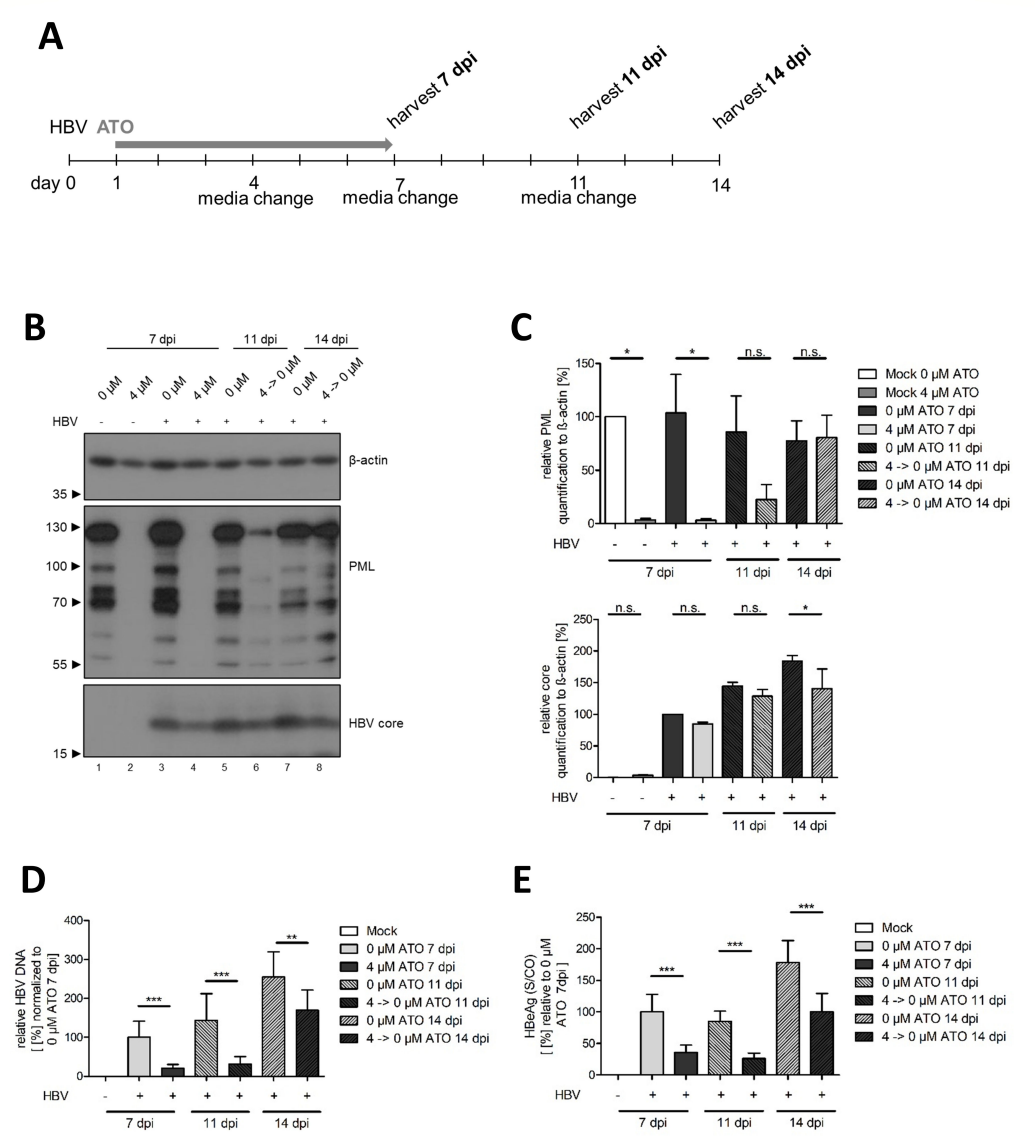
Cessation of ATO treatment induces HBV infection relapse. Differentiated HepG2-NTCP-K7 cells were infected with HBV (MOI 200) and treated with 0 or 4 µM of ATO at 20 hpi. After 7 dpi, the cells were removed from ATO treatment, and the cells were further incubated in a medium without ATO for a further 7 days. Cells were subjected to analysis at 7, 11 (4 days post-ATO cessation), and 14 dpi (7 days post-ATO cessation). (**A**) Schematic representation of the experimental setup. (**B**) Cells were harvested, whole-cell lysates were prepared, separated by SDS-PAGE, and subjected to immunoblotting using mAb 8C9-11 (core), pAb NB100-59787 (PML), and mAb AC-15 (β-actin). Molecular weights in kDa are depicted on the left and respective proteins on the right of each blot. (**C**) Protein expression of the detected bands was quantified by densitometric analysis utilizing ImageJ (Version 1.45 s). Relative protein expression was normalized to the respective β-actin steady-state level. Bar charts represent average values and standard deviations based on two independent experiments. (**D**) Cells were subjected to RT-PCR analysis at 7, 11, and 14 dpi using primers for the total intracellular HBV DNA relative to PRP as an internal control, normalized to untreated, infected cells at 7 dpi. (**E**) Secreted HBeAg quantity in cell culture supernatant was determined by performing an ELISA assay. Results depicted in bar charts represent average values and standard deviations based on six biological replicates. Statistically significant differences were determined using one-way ANOVA. *P ≤ 0.05, **P ≤ 0.01, ***P ≤ 0.001, and ****P ≤ 0.0001.

As treatment of HBV-infected HepG2-NTCP-K7 induced a reduction in cccDNA levels, but never a complete response (Fig. 2B and 4E), we asked the question if the productive HBV infection might relapse upon cessation of ATO treatment. Therefore, HBV-infected HepG2-NTCP-K7 cells were treated with 4 µM of ATO at 20 hpi. At 7 dpi, the ATO treatment was terminated, and cells were cultured for a further 7 days without ATO and analyzed at 7 11 (4 days post-ATO cessation), and 14 dpi (7 days post-ATO cessation). As expected from the literature, the removal of ATO induced a re-appearance of PML protein in western blot (60–63) at 11 and 14 dpi (Fig. 6B and C). At 7 dpi, ATO treatment led to a strong reduction in HBV core protein levels (Fig. 6B and C), total HBV DNA (Fig. 6D), and secreted HBeAg (Fig. 6A), as expected from our previous results. Cessation of ATO treatment led to a gradual re-appearance of HBV parameters, including HBV core protein (Fig. 6B and C), total HBV DNA (Fig. 6D), and secreted HBeAg (Fig. 6E). These parameters, however, never reach the levels of an untreated HBV infection, indicating that ATO treatment for a short time period can significantly delay HBV infection.

### ATO treatment disperses HBV core protein significantly from PML-NBs

**Fig 7:**
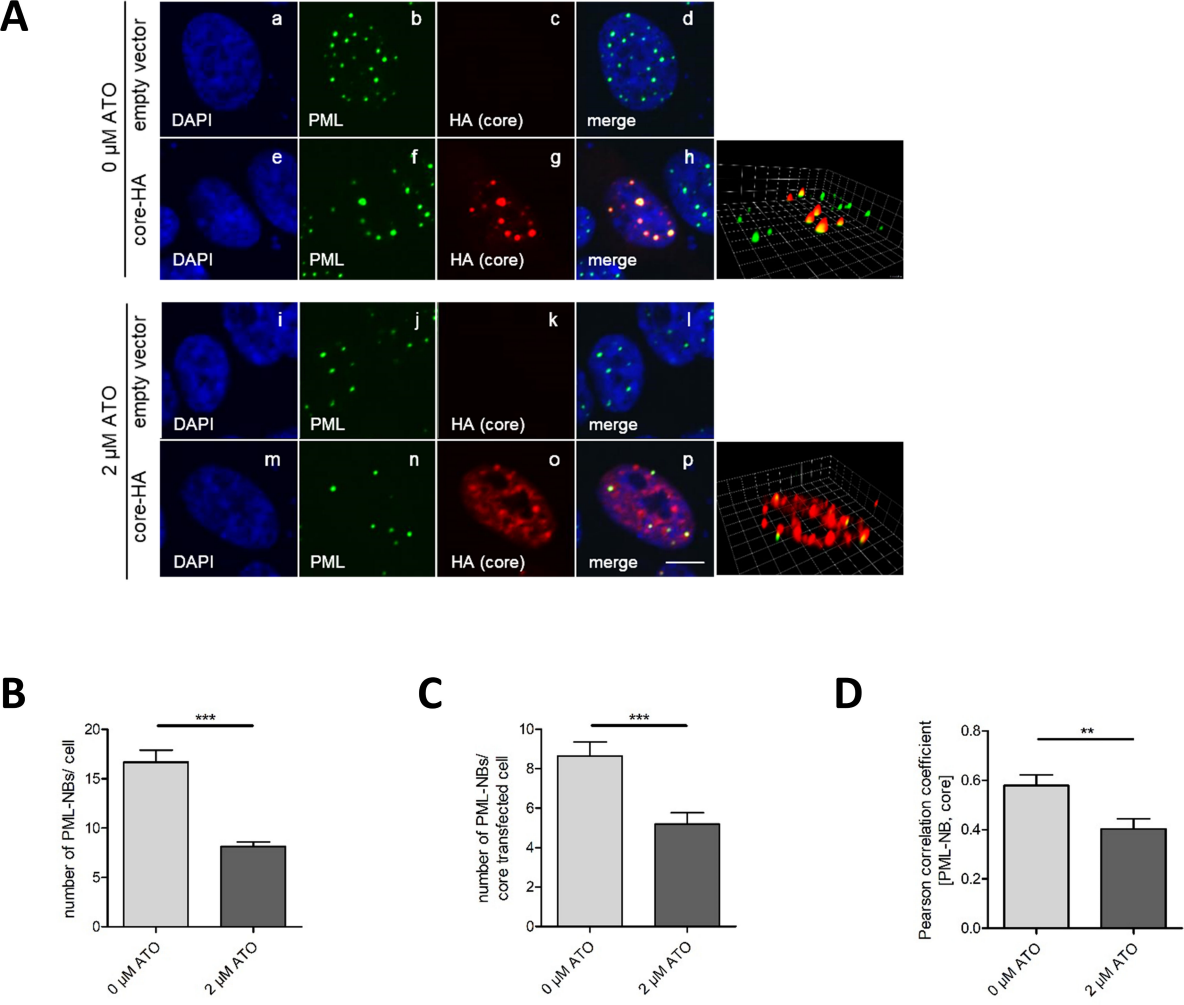
ATO treatment relocalizes transfected HBV core away from PML-NBs. HepaRG His/HA cells were transfected with HBV core-HA and treated with 2 µM ATO at 3 hpt for 48 h. (**A**) HepaRG His/HA cells were fixed with 4% paraformaldehyde and stained with mAb 3F10 (HA) and pAb NB100-59787 (PML). Primary antibodies were detected by conjugated secondary antibodies Alexa488 (PML, green) and Alexa647 (HA, red). Representative pictures as well as Z-stack images are shown. Scale bar represents 7 µm. (**B**) Number of PML-NB per cell in HepaRG His/HA cells with and without ATO treatment. (**C**) Number of PML-NBs in core-HA-transfected HepaRG His/HA cells with and without ATO administration. (**D**) Colocalization of core-HA protein with endogenous PML staining in HepaRG His/HA cells with and without ATO administration was quantified using the Pearson colocalization coefficient via Volocity software.

Finally, we investigated the impact of ATO on the localization of transfected HBV core-HA protein in HepaRG His/HA cells. As previously described by our group, the HBV core protein can be post-translationally modified by SUMO2 and localized to PML-NBs (48). The immunofluorescence stainings showed a decrease in PML-NBs after ATO treatment in HepaRG His/HA cells (Fig. 7A), as shown for HepG2-NTCP-K7 cells (Fig. 3 and 5). To confirm and quantify the efficiency of ATO treatment, the number of PML-NBs in the complete population (Fig. 7B) as well as in core-HA expressing cells (Fig. 7C) was calculated, further validating the reduction of PML-NBs in HepaRG cells during ATO treatment.

Intriguingly, in ATO-untreated cells, transfected core-HA localized to PML-NBs (Fig. 7A, panels e–h) as previously described (48). Application of ATO induced the separation of core-HA from PML-NBs as well as diffuse distribution in the nucleus (Fig. 7A and B, panels m–p). These findings were confirmed by Z-stack images (Fig. 7A, right panels) and quantification of the Pearson correlation between PML and core-HA (Fig. 7D), which showed a significant loss of core-HA localization to PML-NBs during ATO treatment. In summary, ATO treatment led to a significant redistribution of HBV core away from PML-NBs.

## DISCUSSION

Chronic HBV infection is the major risk factor for the development of hepatocellular carcinoma, accounting for 50%–80% of HCC, which is one of the leading causes of cancer-related deaths worldwide (18, 73, 74). Despite the broad availability of an efficient vaccination strategy, a noteworthy proportion of the population is still chronically infected with HBV, mostly due to transmission in early childhood, prior to vaccination (75–78). The main challenge for the achievement of a functional cure for HBV is the cccDNA, the highly stable persistence reservoir of HBV, which is maintained by further rounds of infection with newly generated progeny viruses or by intracellular recycling of mature nucleocapsids (1, 12, 71). Eradication of the cccDNA is considered to be the holy grail for HBV curative treatment; however, current therapeutic approaches fail to directly tackle this HBV persistence reservoir (79). Recent treatment strategies either aim at reconstitution of the exhausted innate and adaptive immune system in chronic HBV or enhance antiviral immunity by stimulation with PEGylated interferon alpha2a (20, 79). Alternatively, they directly interfere with various steps of the HBV life cycle, including viral entry (22–26), inhibition of the reverse transcriptase function of the HBV polymerase by nucleos(t)ide analogs (19, 20), and a novel class of HBV inhibitors interferes with the correct assembly of HBV nucleocapsids by either yielding aberrant capsid structures or empty capsids (21, 79). Despite the capsid assembly modulators being able to block the intracellular amplification pathway of cccDNA, none of the strategies outlined above is able to reduce established cccDNA pools (21, 79), and novel treatment approaches, which, at least partially, tackle the cccDNA are urgently needed.

Known as a highly dynamic molecular hub for several key cellular processes, PML-NBs change their composition depending on the cellular status and cellular stress, mostly due to changes in SUMOylation and subsequent interactions of SUMOylated proteins with proteins harboring a SUMO-interacting motif (SIM) (29). PML-NBs are strongly linked with the host cell DNA damage response and are implicated in the regulation of cccDNA formation (40–43).

ATO, approved since 2000 for the treatment of acute promyelocytic leukemia, directly binds to PML proteins, thereby massively changing the amount of PML-NBs in the cell and probably changing the composition of the remaining bodies and deregulating the SUMO pool of the host cell (48, 51, 56, 60–63). The antiviral activity of ATO was first described for HCV, whereby the authors show a strong inhibition of HCV infection, proposed to be caused by the induction of reactive oxygen species and not by the inhibitory effect of ATO on PML (80).

During prior investigations of our group, we were already able to show the significant impact of ATO treatment on human adenovirus infection, which is proposed to be based on a reorganization of PML-NBs and deregulation of the host cellular SUMO pool (51). Furthermore, our group revealed a distinct role of the post-translational modification of the HBV core protein with the host cellular protein SUMO and the subsequent association of SUMOylated core protein with PML nuclear bodies in cccDNA establishment and maintenance (48). Based on these previous studies, we hypothesize that the treatment of HBV with ATO also interferes with efficient HBV infection and might even affect cccDNA formation and stability.

We could indeed observe a pronounced and efficient reduction in HBV core protein expression with IC50 values of 4.64 and 2.88 µM at 4 and 7 dpi, respectively. Furthermore, we found a decrease in HBV core protein expression, which was confirmed by western blotting and fluorescence-activated cell sorting(FACS) analysis. In addition, a significant decrease in complete DNA and HBV RNA levels, as well as a strong repression of HBeAg secretion, was observed, showing efficient suppression of HBV infection when cells were treated directly after the removal of the inoculum. Furthermore, 4 µM ATO induced a decrease in cccDNA levels of around 30% and 20% at 4 and 7 dpi, showing a decent effect on cccDNA establishment and maintenance; however, no complete remission of cccDNA was detectable. Even when ATO was applied to cells infected with HBV for 3 days prior to treatment, representing a timeframe the HBV cccDNA pool is stably established in HepG2-NTCP-K7 cells (71), a severe and significant reduction in HBV protein expression, HBeAg secretion, HBV transcript levels, and HBV complete DNA was observed. Even under these conditions of established cccDNA, a reduction in cccDNA levels of 30% at 3 dpt was observed, which further developed to a 50% decrease at 6 dpt, both for cells treated with 4 µM ATO. Taken together, these results show a distinct and strong suppression of HBV replication, even under conditions of an established infection, and these promising results might pave the way for the development of a novel antiviral strategy.

We did, however, only observe reductions and never complete removal of HBV cccDNA, which raised the question of HBV relapse upon cessation of ATO treatment. Similar to the nucleos(t)ide analogs lamivudine and entecavir (81, 82), treatment discontinuation of ATO led to a rebound of parameters of ongoing HBV infection, including HBV core protein, HBeAg, and total HBV DNA. ATO treatment could, however, significantly delay the ongoing HBV infection, as the infection in the treated cells did not recover to the levels of untreated HBV-infected cells. These results indicate that stand-alone treatment with ATO might not be able to lead to a complete remission of HBV infection but needs to be combined with already existing and clinically approved HBV therapy options, including PEGylated interferon alpha2a and/or NAs like lamivudine, adefovir, entecavir, telbivudine, or tenofovir (19, 20), which will be part of future research.

We could additionally show distinct changes in the subcellular localization of HBV core protein upon treatment with ATO. A switch from the already published nuclear and cytoplasmic localization in untreated, infected cells (68–70) toward the nuclear exclusion of the HBV core protein and accumulation in the cytoplasm, juxtaposed to the nucleus, when cells were treated with ATO was found, indicating a defect in the nuclear entry. During transfection of HBV core-HA, relocalization of HBV core protein away from PML-NBs could be observed in cells treated with ATO. Taken together, these results strongly resemble the phenotype of SUMOylation-deficient HBV core proteins, as recently published by our group (48). Previously published results showed that the inhibition of HAdV infection by ATO, at least partially, relies on depletion of the host cellular SUMO pool (51). We therefore hypothesize that ATO acts on HBV replication by interference with the SUMO modification of the HBV core protein, inducing the disruption of the association of HBV core protein with PML-NBs and inhibiting nuclear entry of mature rcDNA-containing nucleocapsids (48). This nuclear exclusion and entry defect of HBV core proteins probably disturb the intracellular amplification pathway of cccDNA by reinfection with mature nucleocapsids (15, 16, 71). This mode of action is similar to capsid assembly modulators (21) and might contribute, at least partially, to the reduction in cccDNA by the inhibition of the maintenance of the cccDNA pool. In line with this hypothesis, the cytoplasmic core protein accumulations juxtaposed to the nucleus might represent HBV nucleocapsids stuck in the nuclear pore complex.

Several studies link the dynamically regulated PML-NBs with the DNA damage response (DDR) and indicate that they serve as molecular hubs for the host DDR based on SUMO/SIM interactions (40–43). Based on our findings showing a strong link between HBV core SUMOylation and the subsequent PML association with cccDNA formation and the necessity of PML in this process, we propose a model in which rc- to cccDNA conversion by the host DDR takes place at specific PML-NBs and is orchestrated by PML (48). ATO treatment induces hyperSUMOylation of PML, followed by proteasomal degradation, and therefore reduces and probably also changes the composition of PML-NBs (51, 56, 60–63). The loss of cccDNA during ATO treatment might therefore be caused by a loss of the PML protein itself and additionally by the loss or redistribution of PML-NB-associated factors necessary for cccDNA formation. In line with this hypothesis, it was published that ATO treatment decreases the levels and activity of DNA topoisomerase II, a protein that was shown to be implicated in rc- to cccDNA conversion (83, 84).

HBV core protein was shown to be involved in the transcriptional regulation of HBV cccDNA transcription and to enhance transcription levels by promoting an epigenetically permissive state (84). With the cccDNA residing in the nucleus (85), nuclear exclusion of HBV core protein and therefore loss of core protein from the cccDNA might change the epigenetics of cccDNA to a more tightly regulated and repressed state. This could account for the severe reduction in HBV protein expression, HBeAg secretion, and mRNA transcription observed under ATO treatment, which probably could not be caused by the limited reduction of HBV cccDNA alone. Furthermore, our group could show that cccDNA is interacting with PML-NBs (48). It is therefore possible that loss of PML-NBs and changes in their composition during ATO treatment might further destabilize the cccDNA and change its transcriptional activity. This is further underlined by the mutual re-appearance of PML and parameters of ongoing HBV infection after cessation of ATO treatment, indicating that PML is indeed a proviral entity during HBV infection.

Several studies on the impact of arsenic trioxide, mostly of so-called “arsenicals,” organic metabolites of the inorganic arsenic trioxide, on chronic HBV infection and HCC have reported conflicting results. Two studies determined the effect of elevated arsenic exposure due to drinking water contamination, which is a problem for around 200 million people worldwide (65, 66, 86). Zhang et al. (65) hereby monitored urinary arsenic concentration as a measure of arsenic exposure and found a correlation between elevated arsenic levels and the incidence of chronic HBV infection. However, also a correlation between urinary arsenic concentration and ethnicity and birthplace was found. Hsu et al. (66), however, reported a different result in their study, showing that increased arsenic intake from contaminated drinking water reduced the incidence of liver cancer in HBsAg-positive patients and even led to an increase in the number of inactive HBV carriers. In line with these results, a phase II clinical trial on the impact of ATO on HCC showed a partial response in an HBV-positive patient (87). A further study in HBsAg-positive mice indicated a decreased size and incidence of HCC in ATO-treated mice, however, with no effect on the survival rates (64). Considering our promising results concerning ATO as a novel therapeutic for HBV infections, further studies in human liver chimeric mice with chronic and acute HBV infection might prove ATO as an effective tool in controlling the global HBV pandemic.

**Fig 8:**
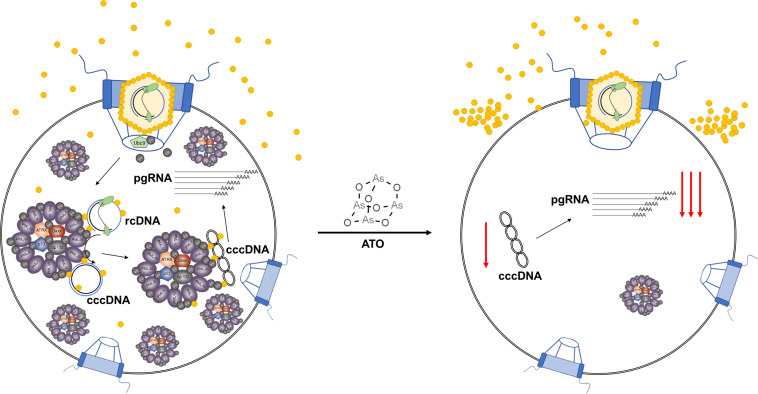
Schematic representation of the proposed mechanism of action of ATO during HBV infection. Our data revealed the reduction of the HBV parameters including the HBV core protein by ATO administration. We presume that the recruitment and/or localization of core to PML-NBs is interrupted during ATO application, resulting in antiviral intervention during novel as well as established HBV infections.

Taken together, our results propose a model for ATO, in which the HBV replication cycle is disrupted at several points. In untreated cells, HBV nucleocapsids are disassembled via SUMOylation of HBV core protein, followed by recruitment of core and the rcDNA to PML-NBs, where cccDNA synthesis takes place (48). The transcriptional permissive state of the cccDNA is then maintained, among others, by the HBV core protein (84). During ATO treatment, we propose that HBV core protein SUMOylation is disturbed, resulting in nuclear exclusion of the core protein, which leads to a defect in the intracellular amplification pathway of cccDNA (15, 16, 71) and loss of transcriptional regulation by core protein. Additionally, the loss of PML-NBs and proteins involved in cccDNA generation enhances the loss of cccDNA (Fig. 8).

These findings represent an important step in the direction of a novel antiviral therapy for chronic HBV infections, which is urgently required since still over 296 million individuals worldwide suffer from HBV, causing more than 820,000 deaths per year (17). In summary, we suggest ATO as a possible antiviral intervention option against HBV by deregulating endogenous host structures, thereby blocking HBV replication and affecting the persistence reservoir, the stable cccDNA.

## MATERIALS AND METHODS

### Cell culture and cell lines

HepG2-NTCP-K7 cells (71) were maintained in Dulbecco’s modified Eagle’s medium (DMEM) supplemented with 10% fetal bovine serum (FBS), 2 mM L-glutamine, 2 mM penicillin/streptomycin, 1 mM sodium pyruvate, and non-essential amino acids (Thermo-Fisher Scientific, Waltham, MA, USA) on collagen-coated cell culture dishes at 37°C in a 5% CO_2_ atmosphere. For HepG2-NTCP-K7 differentiation, 2.5% DMSO was added to the cultivating media. HepaRG His/HA were a kind gift from Prof. Roger Everett (University of Glasgow/UK) and maintained in DMEM containing 10% FBS, 2 mM penicillin/streptomycin, 5 µg/mL bovine insulin, and 0.5 µM hydrocortisone. All cell lines were frequently tested for mycoplasma contamination.

### Arsenic trioxide treatment

Arsenic trioxide (Sigma-Aldrich, St. Louis, MO, USA) was prepared as a 100 mM stock solution in 1 M NaOH. For the treatment of cells, the stock solution was diluted to 1 mM in PBS, and the depicted concentrations were added directly to the cultivating medium of infected or transfected cells.

### Transient transfection and plasmids

pCore-HA encoding HA-tagged HBV core protein, as well as transfection of plasmids using 25 kDa linear polyethylenimine (Polysciences), was recently described (48).

### Cell viability assays

The viability of HBV-infected and ATO-treated HepG2-NTCP-K7 cells was assessed at 4 and 7 days post-infection using the CellTiter-Blue Cell Viability Assay system (Promega, Madison, WI, USA) according to the manufacturer’s instructions. Fluorescence readout was measured using an Infinite 200M plate reader (Tecan, Männedorf, Switzerland).

### Viruses

HBV wt stocks used in this study were kindly provided by the Protzer Lab (Technical University of Munich, Munich), and infection of HepG2-NTCP-K7 cells was performed as recently described (71).

### Protein analysis and antibodies

Whole-cell protein lysates were prepared using RIPA lysis buffer as recently described (46). Lysates were separated by SDS-PAGE, transferred on a nitrocellulose blotting membrane (0.45 µm), and detected via western blotting as previously described (88). As primary antibody for HBV Core protein, mouse mAb 8C9-11 (71), mouse mAb sc-23945 (Santa Cruz), and HBV Capsid rabbit pAb B0586 (DAKO) were used. Cellular protein steady-state levels were determined using polyclonal rabbit Ab raised against PML protein (NB100-59787; Novus Biologicals), and β-actin mouse mAb AC-15 as a control (Sigma-Aldrich, Inc.). mAb3F10 was used for the detection of HA-tagged proteins. To detect proteins by immunoblotting, secondary anti-rabbit IgG and anti-mouse IgG antibodies conjugated to horseradish peroxidase (Jackson/Dianova) were used.

Western blots were processed using Adobe Photoshop CS5 and Adobe Illustrator CS5 software. For relative quantification of protein steady-state levels and comparisons, ImageJ 1.52a (89) was used.

### Indirect immunofluorescence

For the determination of protein localization using indirect immunofluorescence, cells were seeded on glass coverslips, which were additionally coated with collagen for HepG2-NTCP-K7, in 12-well plates at 6 × 10^5^ cells/well. Cells were fixed at indicated time points by 20-min incubation in 4% paraformaldehyde in PBS at RT and stored at 4°C in PBS. Cells were permeabilized in 0.5% Triton X-100 in PBS for 5 min at RT, prior to blocking in tris-buffered saline-BG [TBS-BG; BG is 5% (wt/vol) BSA and 5% (wt/vol) glycine] buffer for 30 min. The coverslips were incubated for 1 h at room temperature with the respective primary antibody, washed three times with PBS, and incubated in the respective Alexa 488 (Invitrogen)- or Alexa 647 (Dianova)-conjugated secondary antibodies overnight at 4°C. DAPI was used for nuclear staining. The coverslips were washed three times in PBS and mounted in Mowiol 4-88 (Carl Roth, Karlsruhe, Germany). Fluorescence images were elaborated using a confocal laser-scanning microscope (Nikon). Image analysis was performed and analyzed using the Volocity software. Colocalization analysis by Pearson correlation was performed using ImageJ 1.52a (89) and the Just Another Co-localization Plugin (90).

### Tecan measurement of core protein expression

HepG2-NTCP-K7 cells were seeded in 96-well plates, differentiated for 3 days, and infected with HBV as described elsewhere (71). At 20 h post-infection, the inoculum was removed, cells were washed twice with PBS, and incubated in a differentiation medium supplemented with ATO in the depicted concentrations for further 3 or 6 days. HBV core proteins were detected by indirect immunofluorescence, whereby the fluorescence intensity was determined utilizing a Tecan Infinite 200M plate reader using an excitation and emission wavelength of 488 and 520 nm for Alexa 488.

### HBV mRNA synthesis

Isolation of mRNA from HBV-infected and ATO-treated cells and reverse transcription were performed as described in reference (91). For quantitative RT-PCR, 4 µL of 1:10 diluted cDNA was supplemented with 10 pmol/µL of the corresponding oligonucleotide primers and 5 µL of SYBR Green Mastermix (Roche) per sample. Relative quantification in relation to the cellular GAPDH mRNA was performed in a LightCycler 480 (Roche) using the following PCR conditions: 10 min at 95°C and 40 cycles of 30 s at 95°C, 30 s at 62°C, and 30 s at 72°C. Technical triplicates were performed for each sample. The primers used for mRNA quantification were (forward and reverse primers): total HBV transcripts (TCACCAGCACCATGCAAC, AAGCCACCCAAGGCACAG) and PRP (TGCTGGGAAGTGCCATGAG, CGGTGCATGTTTTCACGATAGTA).

### Real-time quantitative PCR quantification of HBV DNA

Quantification of total HBV DNA, as well as specific quantification of HBV cccDNA, was performed as recently described elsewhere (71).

### Determination of secreted HBeAg quantity by ELISA

Levels of secreted HBeAg from HBV-infected cells treated with or without ATO were determined as previously published (17).

### Flow cytometry

HBc expression of HBV-infected and ATO-treated HepG2-NTCP-K7 cells at 4- and 7-days post-infection was assessed by flow cytometry. Cells were detached from cultivation plates by trypsin and EDTA (1:1), resuspended in PBS supplemented with 1% FCS, and washed once with PBS. The following steps were performed according to the FIX&PERM (eBioscience) kit using a polyclonal rabbit Ab raised against HBc protein (1:50; CellMarque). Fluorescence readout was measured using flow cytometry (Cytoflex, Beckman Coulter, Brea, CA, USA).

### Statistical analyses

Statistically significant evaluations in mean values were verified by performing one-way ANOVA tests and carried out utilizing the GraphPad (San Diego, CA, USA) Prism5 software.
